# Defining a Path Toward the Use of Fast-Scan Cyclic Voltammetry in Human Studies

**DOI:** 10.3389/fnins.2021.728092

**Published:** 2021-11-12

**Authors:** Suelen Lucio Boschen, James Trevathan, Seth A. Hara, Anders Asp, J. Luis Lujan

**Affiliations:** ^1^Applied Computational Neurophysiology and Neuromodulation Laboratory, Department of Neurologic Surgery, Mayo Clinic, Rochester, MN, United States; ^2^Department of Biomedical Engineering, University of Wisconsin-Madison, Madison, WI, United States; ^3^Division of Engineering, Mayo Clinic, Rochester, MN, United States; ^4^Mayo Clinic Graduate School of Biomedical Sciences, Mayo Clinic, Rochester, MN, United States; ^5^Department of Physiology and Biomedical Engineering, Mayo Clinic, Rochester, MN, United States

**Keywords:** clinical neurochemistry, deep brain stimulation, fast scan cyclic voltammetry, intraoperative, neurochemical signaling, neurophysiology

## Abstract

Fast Scan Cyclic Voltammetry (FSCV) has been used for decades as a neurochemical tool for *in vivo* detection of phasic changes in electroactive neurotransmitters in animal models. Recently, multiple research groups have initiated human neurochemical studies using FSCV or demonstrated interest in bringing FSCV into clinical use. However, there remain technical challenges that limit clinical implementation of FSCV by creating barriers to appropriate scientific rigor and patient safety. In order to progress with clinical FSCV, these limitations must be first addressed through (1) appropriate pre-clinical studies to ensure accurate measurement of neurotransmitters and (2) the application of a risk management framework to assess patient safety. The intent of this work is to bring awareness of the current issues associated with FSCV to the scientific, engineering, and clinical communities and encourage them to seek solutions or alternatives that ensure data accuracy, rigor and reproducibility, and patient safety.

## Introduction

**FIGURE 1:**
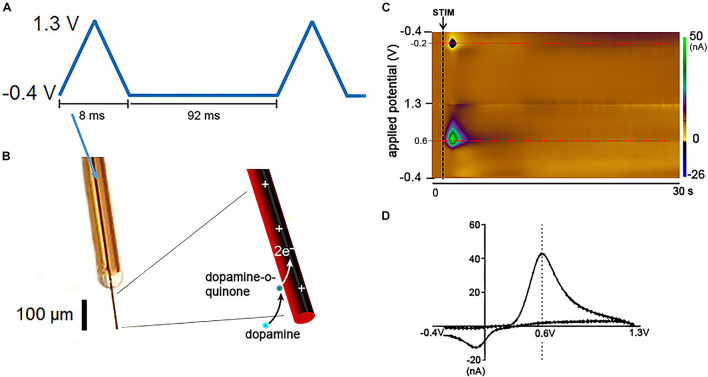
Fast Scan Cyclic Voltammetry in dopamine detection. Schematic of FSCV dopamine detection at CFM. **(A)** Triangular waveform typically used for catecholamine detection. Each waveform ranging from –0.4 to 1.3 V takes 8.0 ms to be delivered and is followed by 92 ms of a constant potential held at –0.4 V. Therefore, each waveform cycle takes 100 ms to be completed. **(B)** Illustration of a silica tubing-insulated CFM with the oxidation/reduction reactions of dopamine at the carbon fiber exposed tip. **(C)** Pseudocolor-plot shows the changes in oxidation (0.6 V) and reduction (–0.2 V) currents (red dashed lines) corresponding to dopamine release evoked by medial forebrain bundle electrical stimulation (black arrow and dashed line) in an anesthetized rat. The oxidation and reduction currents (in nA) are represented in the color scale on the right side of the pseudocolor-plot. The pseudocolor-plot shows 300 triangular waveform cycles (–0.4, 1.3, and –0.4 V) applied from 0 to 30 s at 10 Hz. Additional time after neurotransmitter release is typically showed in pseudocolor-plots to demonstrate that the background current returns to baseline levels. **(D)** Dopamine voltammogram associated with the oxidation and reduction currents represented in the pseudocolor-plot. Adapted with permission from The Schwerdt lab (http://schwerdt.pitt.edu/resources.html).

Fast scan cyclic voltammetry (FSCV) is an electrochemistry technique used for over 30 years to study rapid neurotransmission in the brain of anesthetized and awake and behaving animals (Ganesana et al., 2017; Rodeberg et al., 2017). FSCV detects electroactive neurotransmitters by varying the electric potential between a small working electrode (on the order of a few micrometers in diameter) and a larger reference electrode (on the order of millimeters in diameter). This process oxidizes and/or reduces neurotransmitters at specific potentials and results in electrical currents with amplitude proportional to the concentration of the neurotransmitter in the extracellular space (Clark et al., 2010; Bucher and Wightman, 2015; Figure 1). Under the right conditions, this enables the estimation of changes in neurotransmitter levels with high spatial and temporal resolution, sensitivity, and chemical selectivity (Borland and Michael, 2007; Johnson et al., 2017).

However, *in vivo* neurotransmitter measurement via FSCV can be confounded by several factors including interferant molecules with similar oxidation/reduction potentials to the neurotransmitter of interest, electrode biofouling, shifts in pH and ionic concentrations, or increased oxygenated blood flow in the electrode microenvironment. Additionally, electrical and motion artifacts can disrupt the electrochemical interface creating artificial signals that appear similar to neurotransmitter release (Ariansen et al., 2012; Lee et al., 2017). In small animal studies, a set of guidelines known as the “Five Golden Rules” is typically used to validate and increase reliability of *in vivo* neurotransmitter measurements (Clark et al., 2010). These Golden Rules include (i) identification of neurotransmitter-specific electrochemical signatures, (ii) additional confirmation of the chemical identity of each recorded neurotransmitter (e.g., through microdialysis at the FSCV site), (iii) anatomical validation of the recording location, (iv) kinetic validation of spontaneous or evoked changes in neurotransmitter concentration, and (v) pharmacological validation of recorded neurotransmitters (Clark et al., 2010; Meunier et al., 2018). These guidelines are instrumental toward ensuring the validity of pre-clinical FSCV measurements.

In the last 10 years, there has been a trend toward the use of FSCV in patients undergoing neurosurgical procedures to study human neurophysiology (Bucher and Wightman, 2015). Undoubtedly, advancing the knowledge of underlying mechanisms and biological processes associated with the complex chemistry of the human brain is critical to the development of new and improved therapeutic interventions. For example, the clinical use of FSCV may allow characterization of changes in neurochemical signaling evoked by deep brain stimulation (DBS) and could potentially advance treatment of neurologic diseases (Lee et al., 2017). However, clinical questions that can be currently answered with FSCV are limited by technical barriers that must be addressed to ensure that clinical studies have appropriate scientific rigor and mitigate risks to patient safety. To address potential confounds during data acquisition in humans, extensive pre-clinical testing of new technologies designed to enable human use must be performed. Additionally, there is a critical need for appropriate pre-clinical studies mimicking the clinical environment. Risks should be weighed against actual benefits of the clinical work to ensure patient safety and high-quality data. The objective of this manuscript is to initiate a discussion between the scientific, engineering, and clinical communities regarding these issues and encourage collaborative solutions that are safe and suitable for neurochemical measurements in clinical studies.

## Investigational Use of Fast Scan Cyclic Voltammetry

While a clinical study may not directly benefit the patients participating in the study, studies are likely to improve future patient care. Notably, treatment improvements and benefits to patients can only be accomplished by leveraging appropriate pre-clinical studies and scientific rigor.

### Large Animal Studies

Anatomical and physiological similarities, as well as behavioral correlates to human conditions often make non-human primate and swine models a better alternative for translational research (Lind et al., 2007; Phillips et al., 2014). Unfortunately, only a small number of FSCV studies have investigated neurotransmitter release *in vivo* using large animal models (Table 1).

**TABLE 1:** Fast scan cyclic voltammetry studies in large animals.

**Study**	**Species (common name), *n***	**Goal**	**FSCV parameters**	**FSCV electrodes**	**Major findings**
Cheney-Thamm et al., 1984	*Macaca nemestrina* (Pigtail macaque) 2 females, 2 males	1 – Determine if D-amphetamine increased current oxidation in the caudate of macaques, as previously reported in rodents. 2 – Determine if altered behavioral states induced by D-amphetamine affected oxidation peaks.	Triangular waveform from –0.2 to +0.6 V at 10 mV/s	*Working electrode***:** Teflon coated 250 μm diameter SS wire ∼ 1 mm exposed tip *Reference electrode:* Ag/AgCl wires	D-Amphetamine treatment enhances dopaminergic signaling during presentation of both pleasant and unpleasant stimuli.
Cheney-Thamm et al., 1987	*Macaca nemestrina* (Pigtail macaque) 2 females, 1 male	Demonstrate the feasibility of using acetaminophen as *in vivo* internal standard for electrode calibration.	Triangular waveform from –0.2 to +0.6 V at 10 mV/s	*Working electrode:* Teflon coated 250 μm diameter SS wire *Reference electrode:* Ag/AgCl wire	Acetaminophen peak signal was successfully detected by FSCV in the non-human primate brain.
Earl et al., 1998	*Callithrix jacchus* (marmoset) 8 (unspecified number of males and females)	Evaluate electrically-evoked dopaminergic efflux in the striatum of normal and MPTP-treated marmosets.	Triphasic triangular waveform from –1.0 to +1.4 V and resting potential at 0 V at 480 V/s at 2 Hz (15 ms/scan)	*Working electrode:* Glass insulated 7 μm diameter CFM ∼ 20–50 μm exposed tip *Reference electrode:* Ag/AgCl wire	Dopamine signaling in the striatum remains responsive to MFB electrical stimulation after dopaminergic lesion induced by MPTP.
Shon et al., 2010	*Sus scrofa domesticus* (Swine) 4 males	1 – Investigate if electrical stimulation of the STN evokes striatal dopamine release in a large animal model. 2 – Demonstrate feasibility of performing FSCV recordings in an environment similar to human OR.	Triangular waveform from –0.4 to +1.5 V at 400 V/s at 10 Hz	*Working electrode:* glass insulated CFM (characteristics unspecified) *Reference electrode:* Ag/AgCl wire	STN electrical stimulation evoked intensity and frequency dependent striatal dopamine release.
Lee et al., 2011	*Sus scrofa domesticus* (Swine) Unknown number of animals – males	Use a wireless instantaneous neurotransmitter concentration measurement system to monitor electrochemical signaling in the brain.	Triangular waveform from –0.4 to +1.5 V at 400 V/s at 10 Hz	*Working electrode:* CFM (characteristics unspecified) *Reference electrode:* Ag/AgCl wire	Dopamine signaling responded in sigmoidal-like fashion to pulse intensity and pulse-width STN electrical stimulation.
Ariansen et al., 2012	*Macaca mulatta* (Rhesus macaque) 3 (unspecified number of males and females)	Determine if changes in pH and oxygen are associated with reward and/or reward prediction.	Dopamine – Triangular waveform from –0.4 or –0.6 V to +1.0 or +1.4 V at 400 V/s at 10 Hz. Oxygen – from 0.0 to +0.8 V, a reversal to –1.4 V, and then returned to 0.0 V, at 10 Hz.	*Working electrode:* glass insulated 12 μm diameter CFM with 250 μm exposed tip and coated with Nafion and 4-sulfobenzene; 33 μm coated with Nafion. *Reference electrode:* Ag/AgCl wire	Oxygen and pH changes were associated with the reward and cues that predicted reward. Dopamine responses evoked by reward and cues were overshadowed by pH changes detected by FSCV.
Schluter et al., 2014	*Macaca mulatta* (Rhesus macaque) 2 males	Demonstrate the feasibility of using FSCV to measure real-time changes of dopamine levels in the striatum of macaques.	Triangular waveform from –0.4 to +1.3 V at 400 V/s at 10 Hz	*Working electrode:* fused silica capillary CFM – 125–150 μm exposed tip and 7 μm diameter. *Reference electrode:* Ag/AgCl wire	Demonstrated striatal dopamine responses evoked by VTA/SNc and striatum electrical stimulation, and by unexpected rewards in awake monkeys.
Van Gompel et al., 2014	*Sus scrofa domesticus* (Swine) 3 males	Determine if extracellular adenosine concentration increases during seizure termination (in swine and humans).*	Triangular waveform from –0.4 to +1.5 V at 900 V/s at 10 Hz	*Working electrode:* CFM – 100 μm exposed tip and 7 μm diameter *Reference electrode:* Ag/AgCl wire	Increased adenosine levels were observed just prior to seizure termination. FSCV recordings were also performed in human patients.*
Yoshimi et al., 2015	*Macaca fuscata* (Japanese macaques) 3 females	Measure changes in dopamine levels associated with reward response.	Triangular waveform from –0.4 to +1.3 V at 400 V/s at 10 Hz	*Working electrode:* fused silica CFM and glass capillary CFM – 7 μm diameter and 300 μm exposed tip. *Reference electrode:* Ag/AgCl wire	Dopamine release induced by electrical stimulation and reward signals was detected in the macaque striatum by FSCV on carbon fibers.
Min et al., 2016	*Macaca mulatta* (Rhesus macaque) 3 males	Characterize striatal dopamine release evoked by STN DBS as a function of stimulating and recording electrode location.	Triangular waveform from –0.4 to +1.5 V at 400 V/s at 10 Hz	*Working electrode:* CFM – 7 μm diameter and 100 μm exposed tip. *Reference electrode:* Ag/AgCl wire	Evoked dopamine responses were higher at the stimulation of the dorsolateral posterior border of the STN.
Ross et al., 2016	*Sus scrofa domesticus* (Swine) 17 (unspecified number of males and females)	Determine the functional connectivity between the medial limbic and corticolimbic circuits following fornix DBS via evoked-dopamine release in the NAc.	Triangular waveform from –0.4 to +1.5 V at 400 V/s at 10 Hz	*Working electrode:* CFM – 7 μm diameter and 100 μm exposed tip. *Reference electrode:* Ag/AgCl wire	Electrical stimulation of the fornix induced dopamine release in the NAc and increased BOLD activity in structures along the medial-corticolimbic circuitry.
Lee et al., 2017	*Rattus norvegicus* (Sprague-Dawley rats) 40 males *Sus scrofa domesticus* (Swine) 12 males *Macaca mulatta* (Rhesus macaque) 3 males	Demonstrate proof-of-principle for wireless measurement, characterization, and control of neurotransmitter release.	For dopamine and adenosine – triangular waveform from –0.4 to +1.5 V; for serotonin – N-shaped waveform: –0.4, +1.0, –0.4, +1.4 V	*Working electrode:* CFM – 7 μm diameter and 100 μm exposed tip. *Reference electrode:* Ag/AgCl wire	Demonstrated successful *in vivo*, wireless, single or multi-channel detection of dopamine, adenosine, and serotonin, with integrated sensing and stimulation feedback capabilities.
Nakajima et al., 2017	*Macaca fuscata* (Japanese macaques) 3 females	Evaluate the effects of clinically relevant STN and GPi DBS in the modulation of the activity of tonically active striatal cholinergic interneurons.	Triangular waveform from –0.4 to +1.5 V at 408.6 V/s at 10 kHz for 60 s, and resting potential at 0 V.	*Working electrode:* SS insulated CFM – 250–300 μm exposed tip. *Reference electrode:* Ag/AgCl wire	STN DBS, but not GPi DBS, induced striatal dopamine release that was correlated to increased activity of tonically active cholinergic striatum interneurons.
Schwerdt et al., 2017b	*Macaca mulatta* (Rhesus macaque) 3 females	Demonstrate the feasibility of using an integrated neurochemical modular platform for monitoring dopamine release from sensors chronically implanted in the brain of non-human primates during behavior and stimulation-evoked dopamine release.	Triangular waveform from –0.4 to +1.3 V at 400 V/s at 10 Hz	*Working electrode:* Array of chronic and acute fused silica insulated CFMs – 7 μm diameter and 150–300 μm exposed tip *Reference electrode:* Ag/AgCl wire or SS electrode	Modular platform allowed measurements of dopamine release from multiple sites in the striatum while electrically stimulating the SNc/VTA for up to 170 days.
Settell et al., 2017	*Sus scrofa domesticus* (Swine) 4 (unspecified number of males and females)	Determine the neuromodulatory effects of VTA DBS on dopamine release in the NAc.	Triangular waveform from –0.4 to +1.5 V; or N-shaped waveform: –0.4, +1.0, –0.4, +1.4 V	*Working electrode:* CFM – 7 μm diameter and 100 μm exposed tip. *Reference electrode:* not specified	VTA DBS resulted in increased dopamine release in the NAc and increased BOLD activity in the striatum, cortical and limbic structures.
Trevathan et al., 2017	*Rattus norvegicus* (Sprague-Dawley rats) 12 females *Sus scrofa domesticus* (Swine) 4 males *Macaca mulatta* (Rhesus macaque) 1 male	Characterize subject-specific kinetics of stimulation-evoked dopamine release using computational modeling.	Triangular waveform from –0.4 to +1.5 V at 400 V/s at 10 Hz	*Working electrode:* CFM – 7 μm diameter and 100 μm exposed tip. *Reference electrode:* not specified	Dopamine dynamics in response to electrical stimulation was modeled and characterized based on non-linear increased responses of dopamine to increasing SNc/VTA stimulus intensity.

SS, stainless steel; FSCV, fast scan cyclic voltammetry; MPTP, 1-methyl-4-phenyl-1,2,3,6-tetrahydropyridine CFM, carbon fiber microelectrode; MFB, medial forebrain bundle; STN, subthalamic nucleus; OR, operating room; VTA, ventral tegmental area; SNc, substatia nigra pars compacta; DBS, deep brain stimulation; NAc, nucleus accumbens; BOLD, blood-oxygen-level-dependent imaging; and GPi, globus pallidus internum. *See Table 3.

Dopamine release in large animals was first measured via FSCV in 1984 in a non-human primate model (Cheney-Thamm et al., 1984). In the study, the investigators detected increased current peaks in the oxidation range for dopamine in the caudate in response to pleasant stimuli and amphetamine administration. However, these measurements were not properly validated accordingly to the Five Golden Rules, i.e., use of rudimentary electrochemical parameters and working electrode material, which reduces reliability on the data collected.

It was not until 10 years later that FSCV was used again in non-human primates to demonstrate reduced dopaminergic release in response to electrical stimulation of the medial forebrain bundle in an anesthetized 1-methyl-4-phenyl-1,2,3,6-tetrahydropyridine marmoset model of Parkinson’s Disease (Earl et al., 1998). In this study, investigators validated dopamine measurements by confirming dopamine-specific electrochemical signature, and by demonstrating anatomically, electrically, and pharmacologically evoked changes in the kinetics of dopamine concentration.

Since then, other studies using FSCV in swine and non-human primate models have utilized FSCV recording electrodes (including both working and reference electrodes), recording systems, and analysis techniques that had been well validated in small animal models (Adams, 1976; Stamford et al., 1984; Millar et al., 1985; Suaud-Chagny et al., 1992), ensuring rigor and data reproducibility. Consistently, they have reported increased dopamine release in the caudate-putamen as a function of reward (Yoshimi et al., 2015), and electrical stimulation of the subthalamic nucleus (Nakajima et al., 2017), fornix, and ventral tegmental area (Lee et al., 2017). In addition, FSCV has been used to detect cortical adenosine during seizure termination in swine (Van Gompel et al., 2014), and to serve as the basis of a neurochemical-based closed-loop DBS system in rodent, swine, and non-human primate (Ariansen et al., 2012). More recently, studies in non-human primates proposed new strategies to overcome challenges regarding chronic FSCV recordings in large animals, such as target optimization and spatial resolution (Schluter et al., 2014; Schwerdt et al., 2017b). However, they could not resolve other limitations such as tissue damage, glial encapsulation, electrode biofouling, and material deterioration that caused continuous signal loss (Kozai et al., 2015).

Overall, these pre-clinical studies show the feasibility of recording of neurotransmitters via FSCV in acute and long-term animal models. However, they do not directly address the significant barriers to clinical use of FSCV, especially in the operating room setting.

### Scientific Rigor

It is not possible to apply the Five Golden Rules for neurotransmitter measurement in the human operating room. However, many of the potential confounds for FSCV measurement in clinical studies can be anticipated. Data analysis, including calibration and signal extraction algorithms designed to distinguish neurochemical signals from interferents and noise, depend mainly on the electrochemical properties of the FSCV recording system. For example, the material composition, configuration, and surface properties of FSCV electrodes define the electrochemical properties of the recording system and can affect the recorded signals (Bucher and Wightman, 2015; Ganesana et al., 2017; Johnson et al., 2017; Meunier et al., 2018; Puthongkham and Venton, 2019). Additionally, electrode materials and recording system commonly used in small animal models must be modified to ensure biocompatibility and the ability to reach deep brain targets. Furthermore, confirmation of measured signals accuracy via pharmacologic validation or alternate neurochemical technique in humans is largely not possible in the operating room due to increased hemorrhage risk with additional alternate neurochemical recordings (Sansur et al., 2007; Park et al., 2011), and risk of pharmacological manipulations interfering with patient’s wellbeing during the procedure. Moreover, the time required for recording system stabilization and signal detection is large, but the total allowed operating room time for safe experimental procedures is limited. For example, in acute animal experiments using a single Carbon fiber microelectrode (CFM), identifying an optimal area for recording evoked neurochemical release within a given target region can take several minutes to a few hours. This process requires slowly moving the electrode to different locations within the target and allowing enough time for the signal drift to settle after each movement. In contrast, Institutional Review Boards (IRBs) typically require that the research use of FSCV adds no longer than 15–60 min to the overall procedure, which varies across institutions with an average duration of 4 h. The compressed timeframe of the operating room environment combined with patient safety concerns require the use of one single track, with very little or no time available for electrode repositioning for recording optimization. The operating room environment is also full of sources of noise and interference from electronic equipment that can affect FSCV recordings in unexpected ways. Although not exhaustive, the next sections highlight the need for appropriate pre-clinical studies addressing changes to the recording methods, experimental paradigm, and data analysis techniques beyond what is described in the FSCV literature before human neurophysiology studies can be performed.

### Novel Working Electrodes Designs

**FIGURE 2:**
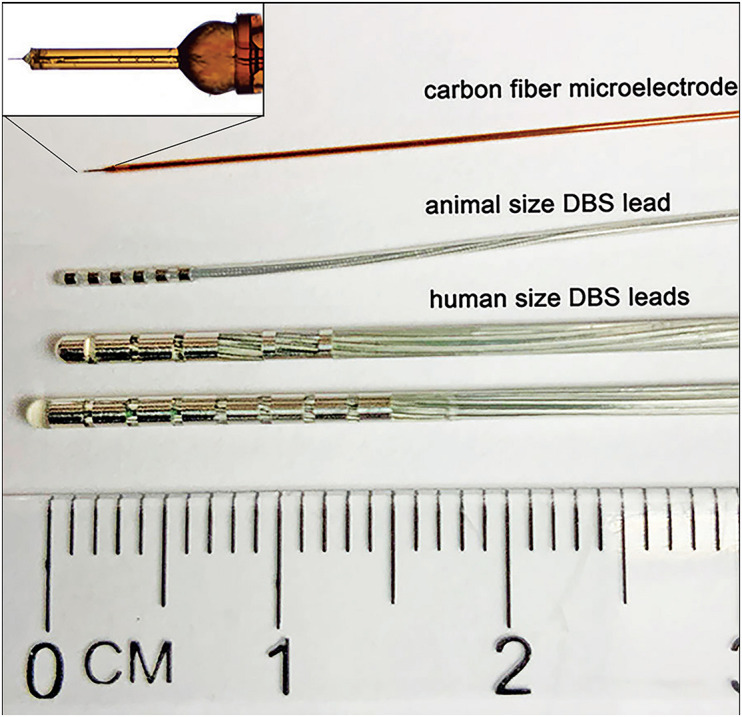
Comparison between typically used FSCV working electrode and DBS electrode leads. From top to bottom: In-house made epoxy and silica tubing-insulated CFM for detection of neurotransmitters via FSCV; Animal size 6-contact DBS electrode lead – NuMED, Inc. (2.0 Fr shaft,0.5 mm spacing, and 42 cm length); Human size DBS electrode leads – Boston Scientific, Inc. (Vercise DBS Directional Lead, 6.0 mm^2^ dome tip contact surface area, 1.5 mm^2^ segmented contacts surface area, 6.0 mm^2^ ring contacts surface area, 1.5 mm contact length, 2.0 mm spacing, 1.3 mm diameter, 30–45 cm length; and Vercise DBS lead, 1.5 mm contact length, 2.0 mm spacing, 1.3 mm diameter, 30–45 cm length).

The working electrode is typically composed of carbon-based materials (Table 2) to improve biocompatibility and chemical inertness, as well as to reduce background currents that interfere with the detection of neurochemical signatures associated with specific neurotransmitters (McDermott and McCreery, 1994; Patel et al., 2013; Rodeberg et al., 2017; Roberts and Sombers, 2018). CFMs have become the standard working electrode for pre-clinical FSCV recordings. The popularity of CFMs can be attributed mainly to their small size, which is critical for minimizing damage to neural tissue and reducing the impact on neurochemical transmission near the electrode (see Figure 2 for CFM and DBS electrode leads size comparison). Similarly, CFM have shown good performance in maintaining normal neurotransmitter kinetics at the measurement site and high adsorption of neurotransmitters of interest (Borland et al., 2005; Wang and Michael, 2012; Jaquins-Gerstl and Michael, 2015). However, despite their desirable neurochemical detection properties, CFMs are not optimal for clinical applications due to their high manufacturing variability (Roberts and Sombers, 2018) and fragility, which increase the risk of breakage during implantation into brain tissue (Schluter et al., 2014). Similarly, surface modified CFM made by coating with polymer films such as Nafion and PEDOT provides enhanced sensitivity, selectivity, and kinetic properties for dopamine detection. However, electrode reproducibility is still poor due to the lack of coating uniformity (Brazell et al., 1987; Pihel et al., 1996; Vreeland et al., 2015; Ganesana et al., 2017; Roberts and Sombers, 2018). In addition, PEDOT is not currently approved for clinical use due to the large range of PEDOT variations determined by multiple factors such as the fabrication technique, the dopant, and the functionalization of the polymer (Boehler et al., 2020).

**TABLE 2:** Characteristics of commonly used fast scan cyclic voltammetry working electrodes in *in vivo* studies.

**Electrode material**	**Type of electrode modification**	**Insulation**	Advantages*	Disadvantages*	**References**
Carbon fiber	N/A	Borosilicate glass	Ease of fabrication, low cost, biocompatible, recommended for acute recordings.	Fragile, high risk of breakage, poor fabrication uniformity, susceptible to fouling.	Patel et al., 2013; Bucher and Wightman, 2015; Rodeberg et al., 2017; Roberts and Sombers, 2018
Carbon fiber	N/A	Fused-silica capillaries sealed with epoxy	Good insulating properties, increased flexibility, low cost, lower risk of breakage, small diameter, biocompatible, recommended for chronic recordings.	Susceptible to fouling, poor fabrication uniformity, torsion applied to the carbon fiber not well translated to the fused-silica insulation.	Clark et al., 2010; Rodeberg et al., 2017; Roberts and Sombers, 2018
Carbon nanotube	Surface-modified CFM	Borosilicate glass or Fused-silica capillaries sealed with epoxy	High mechanical strength, improved electron-transfer kinetics, high sensitivity to adsorbed dopamine	Poor fabrication uniformity and reproducibility.	Bucher and Wightman, 2015; Ganesana et al., 2017; Yang et al., 2017
Gold-nanoparticles	Surface-modified CFM	Borosilicate glass or Fused-silica capillaries sealed with epoxy	High electroactive surface area, fast electron-transfer kinetics, highly sensitive	Increased fouling	Zhao et al., 2012; Mohanaraj et al., 2019
Carbon fiber	Nafion-coated CFM	Borosilicate glass or Fused-silica capillaries sealed with epoxy	Improved selectivity and sensitivity for dopamine, fast response time	Poor uniformity and reproducibility in creating Nafion film around the electrode, fouling susceptibilibility is similar to bare CFM.	Brazell et al., 1987; Pihel et al., 1996; Roberts and Sombers, 2018
Carbon fiber	PEDOT:Nafion-coated CFM	Borosilicate glass or Fused-silica capillaries sealed with epoxy	Improved selectivity and sensitivity for dopamine, fast response time, low fouling	PEDOT is not approved for human use	Vreeland et al., 2015; Ganesana et al., 2017; Boehler et al., 2020
Diamond	Boron doped conductive diamond	Borosilicate glass sealed with resin or Stainless tube sealed with resin or Parylene	Wide potential window, low and stable background current, low fouling, improved longevity and resistance	Low sensitivity, slow electron-transfer kinetics, costly fabrication, poor doping may lead to impurities on the surface	Yoshimi et al., 2011; Patel et al., 2013; Bennet et al., 2016; Ganesana et al., 2017; Chang et al., 2019
Platinum	Metal	Unknown	Less susceptible to breakage, versatile for surface modification and assembly into arrays for multi-neurotransmitter monitoring	Susceptible to corrosion, passivation, increased fouling, low sensitivity.	Jackowska and Krysinski, 2013; Roberts and Sombers, 2018
Gold	Metal	Unknown	Less susceptible to breakage, versatile for surface modification and assembly into arrays or miniature gold electrodes, good sensitivity to catecholamines,	Susceptible to corrosion, passivation, increased fouling, low sensitivity	Zachek et al., 2008; Jackowska and Krysinski, 2013; Roberts and Sombers, 2018

*CFM, Carbon Fiber Microelectrode; PEDOT, poly(3,4-ethylenedioxythiophene).*

*Relative to borosilicate glass CFM.

As an alternative to CFM and surface modified CFM, several groups have attempted to develop Boron-doped diamond (BDD) electrodes, which offer increased strength and longevity compared to CFM (Martin et al., 1998; Suzuki et al., 2007; Yoshimi et al., 2015; Bennet et al., 2016). However, BDD has distinct electrochemical properties and significant characterization studies must be performed to better understand and optimize BDD for neurochemical sensing in the clinical setting. To date, studies have shown that BDD electrodes are not suitable for chronic *in vivo* neurochemical detections due to poor sensitivity and neurochemical adsorption rate, increased fouling, and distorted signals (Yoshimi et al., 2011; Jackowska and Krysinski, 2013; Chang et al., 2019).

Metal electrodes such as Gold and Platinum electrodes provide increased mechanical strength being more resistant to breakage during implantation. Additionally, these materials allow for novel electrode arrays and the capability of multiple neurotransmitter simultaneous detection. However, metal electrodes are hardly used for clinical applications because of the risk of corrosion that can lead to tissue damage, passivation and increased biofouling that interfere with neurotransmitter detection (Zachek et al., 2008; Jackowska and Krysinski, 2013; Roberts and Sombers, 2018).

Although novel electrode materials or configurations, such as Platinum, Gold, and Nafion-coated CFM described above, provide numerous benefits, the representation of signal and artifacts *in vitro* and *in vivo* can vary significantly to those measured with CFMs (Bucher and Wightman, 2015). For this reason, it is important to fully validate novel working electrode designs via large animal experiments with similar signal levels and experimental paradigms to anticipated human studies.

### Reference Electrode Biocompatibility and Stability

Reference electrodes are pivotal to ensure stable, consistent, and neurotransmitter-specific measurements. Reference electrodes should not be polarized (i.e., pulled away from their open circuit potential) during FSCV to avoid sensor drift, low signal-to-noise ratio, and distorted neurochemical signals (Rodeberg et al., 2017). Thus, investigators need to ensure that polarization is avoided by increasing the surface area of the reference electrodes relative to the active CFM, as well as by using materials that have a stable *in vivo* reference potential and low polarizability properties. Reference electrodes for *in vivo* neurotransmitter measurements in animal models are typically made of Ag/AgCl, which are highly cytotoxic making them unsafe for long-term (in the order of hours/days) human use (Hashemi et al., 2011; Kolli et al., 2015; Seaton et al., 2020). To avoid the safety concerns associated with Ag/AgCl electrodes, stainless steel (SS) reference electrodes have been used in humans (Kishida et al., 2011; Bucher and Wightman, 2015). Unfortunately, SS is highly polarizable and has an inhomogeneous grain structure that decreases the stability of its open circuit potential by changing the protein adsorption to the electrode surface, which can shift its open circuit potential (Rajendrachari, 2018). The use of SS reference electrodes for clinical FSCV (see Table 3) is an unresolved confound that can significantly affect measurement sensitivity and specificity. Thus, there is a critical need for the pre-clinical investigation and validation of novel reference electrodes that are not only biocompatible, but also that provide stable reference potentials prior to human use.

**TABLE 3:** FSCV studies in humans.

**Study**	**Goal**	**FSCV parameters**	**FSCV electrodes**	**Major findings**
Kishida et al., 2011	Demonstrate feasibility of sub-second dopamine detection in humans during brain surgery.	Triangular waveform from –0.425 V to +1.380 at 400 V/s at 10 Hz.	*Working electrode*: Glass insulated 7 μm CFM *Reference electrode*: Stainless steel	First demonstration of real-time human dopamine release measured during a behavioral investment task.
Chang et al., 2012	Quantify adenosine concentrations in human subjects with essential tremor during VIM DBS.	Triangular waveform from –0.4 V to +1.5 at 400 V/s at 10 Hz.	*Working electrode*: Glass and polyamide insulated 7 μm CFM with 50 μm exposed tip or silicone insulated 30 μm CFM *Reference electrode*: Stainless steel	Demonstrated changes in extracellular concentrations of adenosine in the VIM of patients undergoing VIM DBS.
Van Gompel et al., 2014	Determine changes in extracellular adenosine concentration during epileptic activity (in swine and humans).*	Triangular waveform from –0.4 V to +1.5 at 400 V/s at 10 Hz.	*Working electrode*: Glass insulated 7 μm CFM *Reference electrode*: Stainless steel, 24 gage	Extracellular adenosine concentration increases prior to seizure termination.
Bennet et al., 2016	Demonstrate sensitivity of diamond-based electrode to detect and quantify neurotransmitter release in human patients undergoing DBS.	Triangular waveform from –0.4 V to +1.5 at 400 V/s at 10 Hz.	*Working electrode*: Parylene-C coated boron-doped diamond, conical shape, 50 μm at the base and 100 μm long. *Reference electrode*: Stainless steel	Dopamine and adenosine release were detected using boron-doped diamond electrode in human patients undergoing DBS for tremor.
Kishida et al., 2016	Determine whether subsecond dopamine fluctuations in the human striatum encode reward prediction errors during a sequential choice task.	Triangular waveform from –0.6 to +1.4 V at 400 V/s at 10 Hz.	*Working electrode*: 7 μm CFM insulated with a polyimide-coated fused-silica capillary *Reference electrode*: Stainless steel	Demonstrated striatal dopamine changes encoding experience-dependent reward prediction error and counterfactual prediction error.
Lohrenz et al., 2016	Compare BOLD activity and dopamine responses during a sequential choice task in humans.	Triangular waveform from –0.6 to +1.4 V at 400 V/s at 10 Hz.	*Working electrode*: 7 μm CFM insulated with a polyimide-coated fused-silica capillary *Reference electrode*: Stainless steel	Demonstrated non-linear relationship between BOLD activity and dopamine release of subjects performing an investment task.
Moran et al., 2018	Demonstrate changes in the serotonergic signaling during a decision making task.	Triangular waveform from –0.6 to +1.4 V at 400 V/s at 10 Hz.	*Working electrode*: 7 μm CFM insulated with a polyimide-coated fused-silica capillary *Reference electrode*: Stainless steel	Striatal serotonin correlates with decisions in a sequential investment game and may encode a strategy that modulates choice selection to mitigate risk.
Montague and Kishida, 2018	Demonstrate a new approach for simultaneous multi-neurotransmitter detection and quantification.	Triangular waveform from –0.6 to +1.4 V at 400 V/s at 10 Hz.	*Working electrode*: 7 μm CFM insulated with a polyimide-coated fused-silica capillary *Reference electrode*: Stainless steel	The elastic net approach extracted a concentration-prediction model for multiple analytes that included dopamine, serotonin, and norepinephrine.

FSCV, fast scan cyclic voltammetry; CFM, carbon fiber microelectrode; VIM, ventral intermediate nucleus of thalamus; DBS, deep brain stimulation; and BOLD, blood-oxygen-level-dependent imaging. *See Table 1.

### Tissue Damage and Signal Integrity

Implantation of both working and reference electrodes disrupts the blood-brain barrier, triggering a cascade of complex molecular and cellular responses such as activation of glial cells, loss of perfusion, secondary metabolic injury, neuronal degeneration, and introduction of mechanical strain, which can affect neurochemical sensing (Woolley et al., 2013; Kozai et al., 2015). For example, studies have shown that brain hemorrhage associated with electrode implantation in the human brain can result in 1–2% of symptomatic hemorrhage (i.e., speech arrest, hemiplegia, agitation, and confusion) and 0.5–0.9% of permanent neurological deficit (Sansur et al., 2007; Park et al., 2011). Therefore, any clinical FSCV work should minimize the risks associated with mechanical tissue damage through rigorous pre-clinical studies aimed at understanding and reducing mechanical damage to neural tissue (ISO10993-6:2016, 2016).

Reduction/oxidation (redox) reactions can also disrupt neurochemical diffusion to the region of the sensing electrode, changing neurochemical concentrations near the electrode and affecting detection of the neurochemical species of interest (Wang and Michael, 2012; Jaquins-Gerstl and Michael, 2015).

Carbon fiber microelectrodes used in pre-clinical studies have traditionally been fabricated from silica glass capillaries or tubing with a small implantation profile (Bucher and Wightman, 2015; Rodeberg et al., 2017). However, clinical FSCV studies have mimicked setups used clinically to implant electrophysiological sensing electrodes and relied on stainless steel cannulas to implant either CFM or diamond electrodes, e.g., FHC#66-IT(AO6) 165 mm SS insertion tube for placing depth electrodes along a trajectory. The effect of the relatively large tissue insult resulting from cannula insertion on measured FSCV signals is not well understood and should be explored in large animal models.

### Spatial Resolution and Heterogeneity of Neurotransmitter Release

The high spatial resolution of FSCV, determined by the size of the working electrode relative to the volume of neurochemical release, is one of the driving motivators for use of FSCV to detect neurochemical signaling. Working electrodes are typically made from 7 to 30 μm diameter carbon fibers cut to 50–500 μm lengths (see Figure 2). It is the small size of the CFM that allows for rapid scan rates (>100 V/s) which enables neurotransmitter measurements on a subsecond timescale without producing large charging currents potentially harmful to the brain tissue (Zachek et al., 2009). The spatial resolution of the CFM is on the order of tens to hundreds of microns (Rodeberg et al., 2017), much higher than microdialysis (Jaquins-Gerstl and Michael, 2015) and functional imaging (Glover, 2011). FSCV high spatial resolution allows for neurotransmitter measurement in discrete brain regions, which could potentially serve as guidance for improved targeting of DBS electrode implantation.

It must be considered that studies have shown inconsistent dopamine kinetics across different microdomains in small and large animal models (Wightman et al., 2007; Moquin and Michael, 2009; Taylor et al., 2015), which can lead to data misinterpretation and demonstrate that FSCV recordings from a single CFM are not adequate for testing specific hypotheses of systemic neurotransmission. Thus, measurements obtained at these high resolutions may not accurately represent activity within the target brain nuclei due to sparse synaptic organization, neuronal heterogeneity, and differences in neurochemical release kinetics throughout the structures of interest. These inconstancies in neurotransmitter release across a target of interest, coupled with limited recording time in the operating room, can make finding a suitable recording site difficult for clinical studies. Sparsity and heterogeneity can be addressed by developing FSCV electrode arrays capable of simultaneously sampling multiple brain regions (Zachek et al., 2010; Patel et al., 2016; Schwerdt et al., 2017a). This, however, comes at the expense of increased risk of hemorrhage and inflammation, which must be weighed carefully against the expected benefit of the study.

### Effects of Sterilization of Fast Scan Cyclic Voltammetry Electrodes on Recorded Signals

Sterilization of implantable devices is paramount to reduce the risk of infection. However, this creates a challenge for FSCV measurements as sterilization procedures can alter the geometrical and chemical surface of the CFM, compromise electrode insulation, affect electrical connections, and ultimately, hinder biocompatibility (Nair, 1995; Godara et al., 2007; Zheng et al., 2011; Chong et al., 2015). For example, CFM working electrodes and Ag/AgCl reference electrodes are composed of heat-sensitive materials, which makes steam and dry heat sterilization inappropriate because these techniques can cause polymer degradation and changes in physical or mechanical properties (Nair, 1995; Mendes et al., 2007). Thus, sterilization procedures should not only be evaluated for sterility prior to clinical use, but also for the effect of surface modification on signal specificity and accuracy.

### Effects of the Operating Room Environment on Recorded Signals

Continuous electrochemical recording is associated with baseline instability and sensor drift as a function of time, ultimately reducing measurement accuracy and reproducibility (DeWaele et al., 2017; Mitchell et al., 2017). This drift can be caused by a myriad of external factors such as temperature fluctuations, changes in the chemical environment, or chemical reactions at the sensing surface (Roberts and Sombers, 2018). The chemical environment can be affected by biofouling caused by extracellular proteins, redox reaction debris, cellular encapsulation around the surface of the electrode, and many other factors (Kachoosangi and Compton, 2007; Patel et al., 2013; Nicolai et al., 2017; Seaton et al., 2020). To minimize these effects, several strategies such as sensor cleaning, recalibration, and even replacement have been used (Hermans et al., 2008; DeWaele et al., 2017; Mitchell et al., 2017). However, these are not feasible in an intraoperative setting, where available time is limited. Additionally, the relatively long period of stabilization following implantation of an FSCV electrode (Ramsson et al., 2015; Rodeberg et al., 2017; Castagnola et al., 2020) limits the time available for data collection in the operating room. Furthermore, the operating room environment has numerous sources of noise. These are known to affect electrophysiological recordings, but their effects have not been characterized for electrochemical measurements using FSCV.

### Challenges for Data Analysis

One of the most challenging aspects of FSCV is the complex and variable nature of the data (Olivieri, 2008; Johnson et al., 2016). Signals obtained *in vivo* often have contributions from multiple analytes that require resolution prior to positive identification and quantification. Consistency and accuracy can be improved with automated multivariate statistical data analyses, such as principal component regression, partial least squares regression, and statistical models. Typically, information across the scan-potential window can be used to separate overlapping signals by using training sets (i.e., signals obtained from electrodes, recording sessions, and/or subjects other than those used for experimental data collection) as calibration models (Johnson et al., 2016, 2017; Kishida et al., 2016; Lohrenz et al., 2016; Rodeberg et al., 2017; Meunier et al., 2018). The effectiveness of these analysis techniques depends on the existence of well-characterized relationships between the potential at the working electrode and the measured redox currents for the neurochemical species of interest. For clinical FSCV, analysis algorithms should be validated *in vivo* using the exact recording setup that will be used clinically, at signal levels comparable to those normally observed in pre-clinical settings.

Recent studies adopted a data analysis algorithm built from a novel statistical model trained on *in vitro* data recorded against Ag/AgCl reference electrodes. However, the algorithm was applied to data recorded using SS reference electrodes in patients during a task performed in the operating room without previous pre-clinical validation (Kishida et al., 2011; Montague and Kishida, 2018). Pre-clinical validation using the Five Golden Rules afore mentioned should be performed prior to clinical use of any analysis algorithms. Thus, algorithms such as the one described above remains largely unvalidated until it is tested both *in vitro* and *in vivo* in a pre-clinical model using the same type of working and reference electrodes as those that will be used clinically.

## Other Considerations and Technical Challenges to Fast Scan Cyclic Voltammetry in Humans

### Patient Safety

Patient safety is paramount whenever clinical studies are performed. As with all clinical studies, the onus of determining safety risk lies with the investigator and local IRB, regardless of whether the study involves the use of a device subjected to an Investigational Device Exemption or not (Code of Federal Regulations, 2021a). While not all risk can be eliminated, it is the responsibility of the investigator and local IRB to create a risk management process in order to identify, isolate, and mitigate as much risk as possible (ISO14971:2019, 2019). In this way, not only is the well-being of the patient protected, but also is the integrity, rigor, and reproducibility of the collected data and resulting conclusions.

### Material Biocompatibility

Any material or device that comes into contact with tissue, especially neural tissue, in a clinical study should be evaluated for biocompatibility. Although extensive discussion of biocompatibility testing is outside of the scope of this work, guidance for this biocompatibility testing is provided by the ISO 10993 standard (ISO10993-1:2018, 2018). It is important to note that even if the material itself is biocompatible, there is a risk of harmful substances to be present on the material as byproducts of manufacturing or other processing steps. In commercially marketed medical devices, these risks are mitigated by manufacturing under Good Manufacturing Practice protocols (Code of Federal Regulations, 2021b), which help control any deviation between manufacturing batches. Currently, no medical device suppliers provide electrodes for FSCV, so it falls on the investigator to ensure consistency between materials tested for biocompatibility and those that are used for clinical FSCV work.

### Electrochemical Reactions Due to Waveform Selection

Different electrode materials exhibit unique electrochemical properties that may lead to changes in the waveforms that are used for neurochemical detection. These waveforms, in turn, affect the various safety considerations listed above. Thus, testing of the electrical FSCV potential waveforms is required to determine the range of voltages that can be safely applied to the tissue without inducing excitotoxicity, electrolysis, and reduced electrode longevity. For example, harmful reactive oxygen species are generated when oxygen is reduced (negative potentials) or when water is oxidized (positive potentials; Roberts and Sombers, 2018). However, the potential at which these redox reactions occur varies depending on the water window for the specific electrode material. For voltage-controlled applications such as FSCV, the water window informs whether a particular waveform will result in electrolysis, which is damaging to tissue (Cogan et al., 2004; Cogan, 2008). Therefore, all FSCV waveforms used should be evaluated for tissue damage and safety for each electrode material and configuration should be stablished.

**FIGURE 3:**
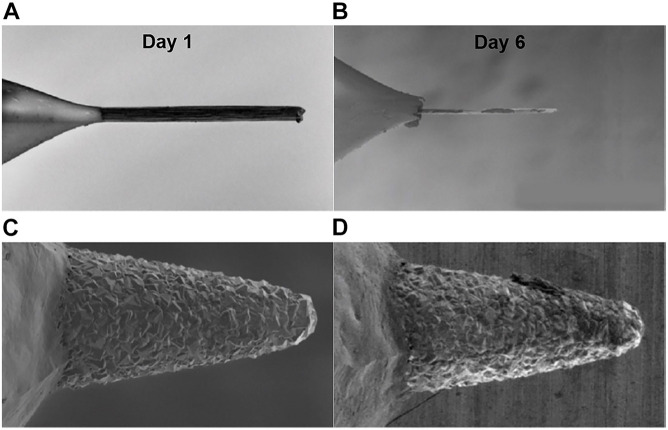
Impact of FSCV waveform on CFM and BDD electrodes longevity. Scanning electron microscope images demonstrate the surface erosion of CFMs **(A,B)** and BDDs **(C,D)** on day 1 **(A,C)** and day 6 **(B,D)** of continuous FSCV. CFMs were significantly eroded after 6 days of continuous FSCV while BDD electrodes show no noticeable changes. A FSCV triangular waveform from -0.4 to 1.5 V and back to -0.4 V at 10 Hz at 400 V/s was continuously applied to CFMs and BDDs immersed in Tris buffer over an extended period of time. Adapted from Bennet et al. (2016).

It is also vital to ensure that the waveform does not damage the electrode itself, as material dissolution is detrimental to the longevity of the electrode (Figure 3) and the released particulate could be harmful to tissue. To prevent electrode damage, the largest potentials of a waveform should be applied to the electrode in solution in a closed-system benchtop setup over an extended period of time and optionally, at a higher frequency than planned. The beaker solution should be sampled at regular intervals and can be tested with mass spectrometry to determine whether the electrode material is dissolved into solution (Boehler et al., 2020). Subsequent biological tests should be conducted to determine whether the intended waveforms would damage cells due to excitotoxicity by conducting safety tests of electrical stimulation in neural tissue (McCreery et al., 1988, 1990, Robblee and Rose, 1990; Shannon, 1992). Finally, pre-clinical studies with thorough histological evaluation of tissue adjacent to and in the vicinity of the implanted electrode should be conducted prior to human studies (Cogan et al., 2004, 2016).

### Sterilization Validation

As would be expected for any invasive medical device, sterilization validation is essential to patient safety (ISO11737-1:2018, 2018). Balancing this need with that of ensuring electrode integrity may require exploration of novel sterilization methods. As previously discussed, the heat-sensitive nature of some of the components commonly used for FSCV electrodes, steam or dry heat sterilization methods are not viable (Nair, 1995; Mendes et al., 2007). However, sterilization methods that do not rely on heat pose other challenges to patient safety. For example, γ radiation generates free radicals that can cause tissue damage (Ries et al., 1996; Gorna and Gogolewski, 2003; Mendes et al., 2007). Similarly, ethylene oxide and its derivatives have been reported to accumulate in some materials, potentially increasing teratogenicity risk (Lucas et al., 2003; Mendes et al., 2007). As previously mentioned, the investigator should characterize and validate each sterilization technique for each FSCV sensor type, composition, and geometry to ensure patient safety, in addition to signal accuracy.

## Discussion

Many of the safety and efficacy concerns associated with FSCV recordings in humans are due to interrelated technical challenges, which makes these challenges difficult to address. However, once the scientific and clinical benefits of the study are clearly defined and the risks to patient safety minimized, residual risks can be weighed against anticipated benefits. This risk-benefit analysis is crucial to the success of the clinical work.

Investigators should perform a compelling risk/benefit analysis to justify the clinical use of FSCV, ensure scientific rigor, and articulate the scientific and clinical benefit of FSCV studies. Addressing and mitigating risk to patient safety will help ensure patients are not put at undue risk. Of course, not all risk can be eliminated, and it is this remaining risk that must be weighed against the proposed clinical benefit. Furthermore, it is essential that both risks and benefits are appropriately and timely communicated to patients through consent forms so patients can make an informed decision about their participation.

Risk-benefit analyses are complex, so the authors propose that the FSCV community develop the necessary guidelines through partnership with the FDA to engender high-quality scientific discoveries that FSCV can offer.

Clinical studies need not demonstrate that FSCV provides direct patient benefit. However, evidence from pre-clinical studies should be used to guide approval for use in human subjects. Application of FSCV in clinical studies should be carefully evaluated to ensure that only hypothesis-driven studies that cannot be appropriately and methodically performed in animal models are conducted in human subjects. Thus, translational FSCV research should demonstrate scientific rationale, feasibility, efficacy, and safety prior to attempting clinical application.

While progress has been made in the clinical use of FSCV (Table 3), it must be stressed that electrode safety and data robustness have yet to be characterized. To date, clinical FSCV studies have employed working and reference electrodes and signal processing techniques that have not been validated by translational research. The Five Golden Rules are not directly applicable in the clinical setting. Thus, the operating room environment necessitates the reconsideration of human FSCV methodologies. Specifically, the following processes need to be performed: acute and chronic target assessment and confirmation, standardization of electrode manufacturing processes, signal verification in human recordings, and characterization of sources of electrical noise in the intra-operative environment. Addressing these issues will facilitate application of FSCV in the clinical setting, particularly if FDA-approved working and reference electrodes become commercially available.

**FIGURE 4:**
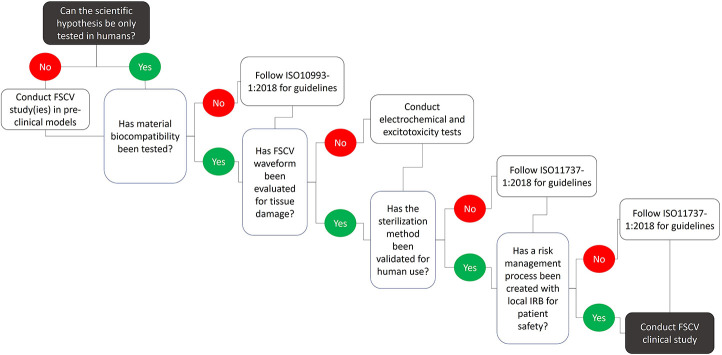
Technical considerations and recommendations for conducting FSCV studies in humans. Numerous limitations associated with FSCV limit the type and number of scientific questions that can be answered by clinical FSCV studies. These limitations pose safety risks to the patient and need to be addressed prior to the clinical use of FSCV. Extensive pre-clinical testing including material biocompatibility, waveform safety, tissue damage, and sterilization methods must be conducted following specific guidelines in order to develop a risk management process in cooperation with local IRBs to establish patient safety.

The use of FSCV in humans must be understood as a clinical intervention, and as such, should follow the same protocols and guidelines required by the FDA to establish device safety (Figure 4). Additionally, there are many gaps in knowledge that complicate the risk/benefit analysis for the clinical use of FSCV during existing surgical procedures. Until these gaps are addressed, the likelihood of obtaining useful scientifically rigorous data is low due to all the unknowns and the quality of the recorded signal in the operating room environment. Thus, clinical FSCV should only be performed when there is a clear hypothesis that can further neuroscientific understanding in the pursuit of improved therapeutic interventions, and only after all risk factors discussed in this manuscript have been mitigated.

## Author Contributions

SLB, JT, SH, and JLL contributed to the conceptualization and design of this work. SLB, JT, and SH wrote the first draft. AA wrote sections of this work. SLB was responsible for the project administration and supervision. JLL supervised the project. All authors contributed to manuscript revision, read, and approved the submitted version review and editing.

## Conflict of Interest

We declare that the following investigators Kenneth T. Kishida, P. Read Montague, Charles D. Blaha, Michael Heien, Kendall H. Lee, Terry Lohrenz may present conflict of interest with the ideas presented in this review because of their past or current use of fast scan cyclic voltammetry in humans. The authors declare that the research was conducted in the absence of any commercial or financial relationships that could be construed as a potential conflict of interest.

## Publisher’s Note

All claims expressed in this article are solely those of the authors and do not necessarily represent those of their affiliated organizations, or those of the publisher, the editors and the reviewers. Any product that may be evaluated in this article, or claim that may be made by its manufacturer, is not guaranteed or endorsed by the publisher.
